# Neuraminidase 1 deficiency attenuates cardiac dysfunction, oxidative stress, fibrosis, inflammatory via AMPK-SIRT3 pathway in diabetic cardiomyopathy mice

**DOI:** 10.7150/ijbs.65938

**Published:** 2022-01-01

**Authors:** Zhen Guo, Hu Tuo, Nan Tang, Fang-Yuan Liu, Shu-Qing Ma, Peng An, Dan Yang, Min-Yu Wang, Di Fan, Zheng Yang, Qi-Zhu Tang

**Affiliations:** 1 Department of Cardiology, Renmin Hospital of Wuhan University, Wuhan 430060, RP China; 2 Hubei Key Laboratory of Metabolic and Chronic Diseases, Wuhan, RP China; 3 The Affiliated Suqian Hospital of Xuzhou Medical University, Suqian 223800, RP China; 4 People's Hospital affiliated to Nanjing Drama Tower Hospital Group, Suqian 223800, RP China; Z.G, H.T, N.T, and FY.L contributed equally.

**Keywords:** Neuraminidase 1, Diabetic cardiomyopathy, SIRT3, LKB1, AMPKα

## Abstract

Diabetic cardiomyopathy (DCM) is associated with oxidative stress and augmented inflammation in the heart. Neuraminidases (NEU) 1 has initially been described as a lysosomal protein which plays a role in the catabolism of glycosylated proteins. We investigated the role of NEU1 in the myocardium in diabetic heart. Streptozotocin (STZ) was injected intraperitoneally to induce diabetes in mice. Neonatal rat ventricular myocytes (NRVMs) were used to verify the effect of shNEU1 *in vitro*. NEU1 is up-regulated in cardiomyocytes under diabetic conditions. NEU1 inhibition alleviated oxidative stress, inflammation and apoptosis, and improved cardiac function in STZ-induced diabetic mice. Furthermore, NEU1 inhibition also attenuated the high glucose-induced increased reactive oxygen species generation, inflammation and, cell death *in vitro*. ShNEU1 activated Sirtuin 3 (SIRT3) signaling pathway, and SIRT3 deficiency blocked shNEU1-mediated cardioprotective effects *in vitro*. More importantly, we found AMPKα was responsible for the elevation of SIRT3 expression via AMPKα-deficiency studies *in vitro* and *in vivo*. Knockdown of LKB1 reversed the effect elicited by shNEU1 *in vitro*. In conclusion, NEU1 inhibition activates AMPKα via LKB1, and subsequently activates sirt3, thereby regulating fibrosis, inflammation, apoptosis and oxidative stress in diabetic myocardial tissue.

## Introduction

Diabetic cardiomyopathy (DCM) is more common than clinically recognized. It is an important cause of heart failure in the world, and an essential burden of medical expenses in the whole country. Epidemiological studies have recognized that diabetes is an independent risk factor for cardiac dysfunction, characterized by myocardial hypertrophy and diastolic/systolic dysfunction 1. The pathophysiology of DCM is complex and despite significant advances, many fields are still not well understood. DCM has a complex pathogenic basis and is therefore difficult to treat by targeting a single candidate mechanism. In briefly, the mechanism of DCM involves increased oxidative/nitrosative stress 2, abnormal calcium signals 3, disordered glucose/fatty acid metabolism 4, 5 and inflammatory pathways^6^ lead to a variety of abnormal biochemical pathways, such as myocardial fibrosis^7^, stiffness and hypertrophy 8, leading to potential pathological changes. These clinical effects develop from asymptomatic diastolic dysfunction to systolic dysfunction and clinical HF.

Neuraminidases (NEUs) are glycosidases, also known as sialidases, that catalyze the removal of α-glucoside-linked sialic acid residues from carbohydrate groups of glycoproteins and glycolipids. Four species of NEUs have been identified according to their subcellular localization and enzymological characteristics, namely NEU1, NEU2, NEU3, and NEU4. In addition to the typical catabolism function in lysosomes, NEU1 can also be transported to the cell surface and participate in the structure and function regulation of cell receptors. Some examples of receptors and cell signaling modulated by NEU1 salivation on the membrane include TLR4-NFκ B-related responses insulin receptors associated with glucose uptake in macrophage phagocytosis Fc receptors for immunoglobulin G, and epidermal growth factor receptors and insulin-like growth factor-2 during proliferation. Compared with healthy controls, increased neuraminidase activity in the plasma of patients with myocardial infarction (MI) has been described 9. NEU1 is involved in the regulation of various cellular metabolic behaviors and signal transduction. Increased NEU1 expression can promote the expression of inflammatory factors (TNF-α, IL-1, IL-6, etc.). The expression of Neu5Ac, a metabolite downstream of NEU1, was increased in patients with coronary heart disease 10. However, the role of NEU1 in DCM has not been reported.

Here, the aims of the present study were 1) to determine whether NEU1 is involved in the pathological process of diabetic cardiomyopathy, 2) to identify how does NEU1 affect the development of DCM, and 3) the specific molecular mechanism of NEU1 affecting DCM.

## Material and methods

### Materials

Streptozotocin (STZ) was purchased from Sigma (St Louis, MO, USA). AMPK si*RNA* and scrambled si*RNA* were obtained from DesignGene Biotechnology (Shanghai, China). 2, 7-Dichlorofluorescin diacetate (DCFH-DA) was ordered from Nanjing Jiancheng Bioengineering Institute (Nanjing, China). Primary antibodies were purchased from Cell Signaling Technology (Danvers, MA, USA), Abcam (Cambridge, UK) and Proteintech (Wuhan, China). Secondary antibodies were purchased from LI-COR Biosciences (Lincoln, NE, USA). The cell counting kit-8 (CCK-8) was obtained from Dojindo Laboratories (Kumamoto, Japan). All other chemicals were of analytical grade.

### Animals

C57BL/6J mice were purchased from the institute of Laboratory Animal Science, Chinese Academy of Medical Sciences (Beijing, China). The source of AMPKα global knockout mice has been described previously 11. Eight to ten-weeks-old (body weight ranging from 20-25g), germ-free male mice were used for the experiments as indicated. All mice were controlled under the condition of 12h light/dark cycle at 21℃ in standard caging and free to diet. All animals' experimental procedures were approved by the Animal Care and Use Committee of Renmin Hospital of Wuhan University.

Animal models constructed on C57BL/6J mice and genetic disrupted AMPK ^-/-^ mice underpin mechanist evaluation of the diabetic cardiomyopathy. Myocardial injection of adeno-associated virus (AAV9, [1×10^10^ vp (viral particles) per animal]) was performed four weeks before STZ injection, and then randomly assigned to STZ injection group or control group. As previously described^12^, ^13^, Diabetic cardiomyopathy model was induced by intraperitoneal (I.P.) injection of streptozotocin (STZ, Sigma, St. Louis, MO) at the dose of 50 mg/kg dissolved in 100 mM citrate buffer pH 4.5 for 5 consecutive days. Control mice received same volumes of citrate buffer. One week following injections, levels of blood glucose (caudal vein) were measured using a Glucometer (Johnson & Johnson, USA). Mice with glucose level >300 mg/dL were considered as having diabetes and were used for further studies 12, 13. AMPK^ -/-^ mice received the same treatment as described above. Blood samples were collected to evaluate the glucose levels and serum biomarkers. And then mice were euthanized with an overdose of sodium pentobarbital (200mg kg^-1^; i.p.) and the hearts were collected.

### Echocardiography and haemodynamic evaluation

Mice were anesthetized by inhalation of 1.5-2% isoflurane. Echocardiography was performed to evaluate the structure and function of the left ventricle using a MyLab 30CV system (Biosound Esaote, Inc.) equipped with a 15-MHz probe. To measure the LV end-diastolic dimension (LVEDD), and LV fractional shortening (FS), M-mode tracings derived from the short axis of the left ventricle at the level of the papillary muscles were recorded; parameters were obtained from at least three beats and averaged. For the haemodynamic analysis, a 1.4-French catheter-tip micromanometer catheter (SPR-839; Millar Instruments, Houston, TX, USA) was inserted into the left ventricle via the right carotid artery to obtain invasive haemodynamic measurements. An aria pressure volume conductance system (MPVS-300 Signal Conditioner, Millar Instruments, Houston, TX, USA) coupled with a PowerLab/4SP A/D converter was used to continuously record the heart rates, pressure, and volume signals.

### Western Blot

Heart tissues and cultured cardiomyocytes were collected and lysed by RIPA containing phosphatase inhibitors PhosSTOP^TM^ tablets and protease inhibitor cocktail cOmplete mini tablets (Roche). Total protein exacted from the heart tissues and cardiomyocytes was separated by 12% SDS-PAGE and then were blotted onto PVDF membranes. The membranes would be blocked in 5% skimmed milk diluted in Tris-buffer saline added Tween-20 (TBST) for 1h at room temperature (RT) before incubating with the primary antibodies. The membranes were thoroughly washed in TBST and then incubated with the primary antibodies at 4℃ overnight. And probed with secondary antibodies for 1h at RT after washing the membranes for the second time. The membranes were washed for the last time and incubated with ECL substrate. All the proteins were normalized to GAPDH and the bands were quantified using an image analysis system (Image Lab). The primary antibodies used are listed in Supplementary Table 1.

### Real-time PCR

1μg total mRNA exacted from the heart tissues and cardiomyocytes using TRIzol regent was worked as the template for the synthesis of cDNA using the Transcriptor First Strand cDNA synthesis kit (Roche). PCR reaction were performed using LightCycler 480 SYBR Green Master Mix and the quantity of the PCR product was normalized to GAPDH. The primers used are presented in Supplementary Table 2.

### Determination of insulin content in pancreas

Briefly, the mice were sacrificed at the specified time point. The pancreatic tissue was carefully removed, and immediately frozen in liquid nitrogen. The samples were homogenized with acidified ethanol (75% ethanol, 1.5% 12 mol/ L HCl and 23.5% H2O), incubated at 4℃ for 72 h, and centrifuged. Then according to the manufacturer's instructions for experimental determination (Beyotime Biotechnology, https://www.beyotime.com/product/PI602.htm).

### Histological analysis, Tunel and Immunohistochemistry staining

Picrosirius red (PSR), HE staining, Tunel staining and immunohistochemical staining were performed following previously described 13, 14. Heart tissues collected from all studied groups were fixed with 10% neutral formalin buffer overnight and embedded with paraffin. The hearts were dissected into 5μm slices. PSR staining was performed to evaluate cardiac fibrosis. HE staining was used to assess the morphological changes of the hearts. All the sections were observed and photographed using a light microscope and a Nikon photoimaging System (Tokyo, Japan) at the magnification of 40× and 200×. To detect cardiac cell death, terminal deoxynucleotidyl transferase dUTP nick-end labelling (TUNEL) staining was used to detect *in situ* detection of apoptosis in diabetic hearts, according to the instructions of ApopTag Plus *in situ* Apoptofluorescein Detection kit (Millipore, MIT, USA). After staining, the slices were observed under the OLYMPUS DX51 fluorescence microscope. The images obtained were blindly analyzed by digital analysis software (Image-Pro Plus 6.0).

For immunohistochemistry, the slices were baked in the oven for over 30min and deparaffined in Xylene 3 times for 15min. Before hyperthermia antigen retrieval with sodium itrate buffer (pH 6.0), the slices were hydrated in gradient ethanol (from 100% to 70%). To eliminate the influence of endogenous peroxidases, slices were incubated with 3% H_2_O_2_ for 10min at RT and then blocked with 8% goat serum for an hour. Sections were stained with anti-TNFα (Cell Signaling Technology, 11948S), anti-CD45 (ABCAM, ab10558), anti-CD68(ABCAM, ab125212) and anti-cleaved-caspase3 (Cell Signaling Technology, 9664,) at 4℃ overnight and incubated with EnVisionTM+/HRP reagent at 37℃ for an hour. To visualize the slices, DAB reagent was used at RT. After that, the slices were mounted with neutral gum and examined using light microscope. For each section, 4-8 visions were counted and a mean value was obtained.

For immunofluorescence staining, paraffin-embedded sections of mice heart tissue were sealed with 8% sheep serum after washing in PBS, and incubated at 4 °C overnight with mouse anti-αSMA primary antibodies (ABCAM, ab5694). After being washed in PBS, the slices were incubated with the secondary antibody shown at room temperature for 1 hour, and then washed with PBS. The nucleus was stained with 4pyr6-diamino-2-phenylindole (DAPI), Invitrogen, S36939). Fluorescence microscope (OLYMPUS DX51) and DP2-BSW software (version 2.2) were used to obtain images.

### Determination of lipid peroxidation, SOD activity and GSH/GSSG content

To further assess oxidative stress level, myocardial malondialdehyde (MDA) or 4 hydroxynonenal levels (4-HNE) levels, SOD activities and glutathione (GSH) oxidized glutathione (GSSG) levels in the myocardial tissues were determined using commercially available kits procured from Beyotime Co. (Nantong, China) according to previous study^15^.

### Cell culture and treatments

Neonatal rat ventricular myocytes (NRVMs) were separated from newborn rat within 3 days and cultured with Dlbecco's modified Eagle's medium (DMEM) added 10% fetal bovine serum (FBS) at 37℃ in incubator gassed with 5% carbon dioxide. When 60% -70% fusions is reached, NRVMs are exposed to normal glucose (NG, 5.5mmol/l) or high glucose (HG, 33mmol/l) respectively at a specified time point. To investigate the protection of SIRT3 *in vitro*, NRVMs were transfected with 100 nmol/l SIRT3 small interfering (si*SIRT3*) or a control nonspecific si*RNA* for 4h^16^. AMPKα knockdown was achieved by si*AMPKα* transfection using Lipofectamine RNAiMAX (Invitrogen) for 24 h, whose efficiency has been proved in our previous study 14, 17. To explore the mechanism through shNEU1 activated AMPKα, NRVMs were incubated with STO-609 (the CaMKK inhibitor, 800 nmol/l) 18, Takinib (the selective TAK1 inhibitor, 10 mM) 19. To knock down LKB1, si*LKB1* was used. To induce cell death, NRVMs were subjected to H_2_O_2_ (200μmol for 12h) treatment.

### Detection of ROS and cell viability

NRVMs which were seeded in 6-well plates were exposed to HG for 24h. To assess the ROS level, the cells were incubated with DCFH-DA for 0.5h (37°C), and then were visualized in a blinded manner under an Olympus IX53fluorescence microscope. Cell count analysis (CCK-8; Dojindo Molecular Technologies, Rockville, MD, USA) was performed according to the manufacturer's protocol assays for cell viability.

### Cardiac ELISA test kits

The concentrations of NEU1 in cardiac homogenates were measured by enzyme-linked immunosorbent assay (ELISA) methods.

### Statistical analysis

All data were expressed as mean ± standard error of mean (SEM). Comparisons between two groups were performed using Student's t test. Differences among groups were assessed using a one-way analysis of variance (ANOVA) followed by Tukey's post hoc test. Probability values of *P*<0.05 were considered significant.

## Results

### NEU1 is up-regulated in cardiomyocytes under diabetic conditions

**Figure 1:**
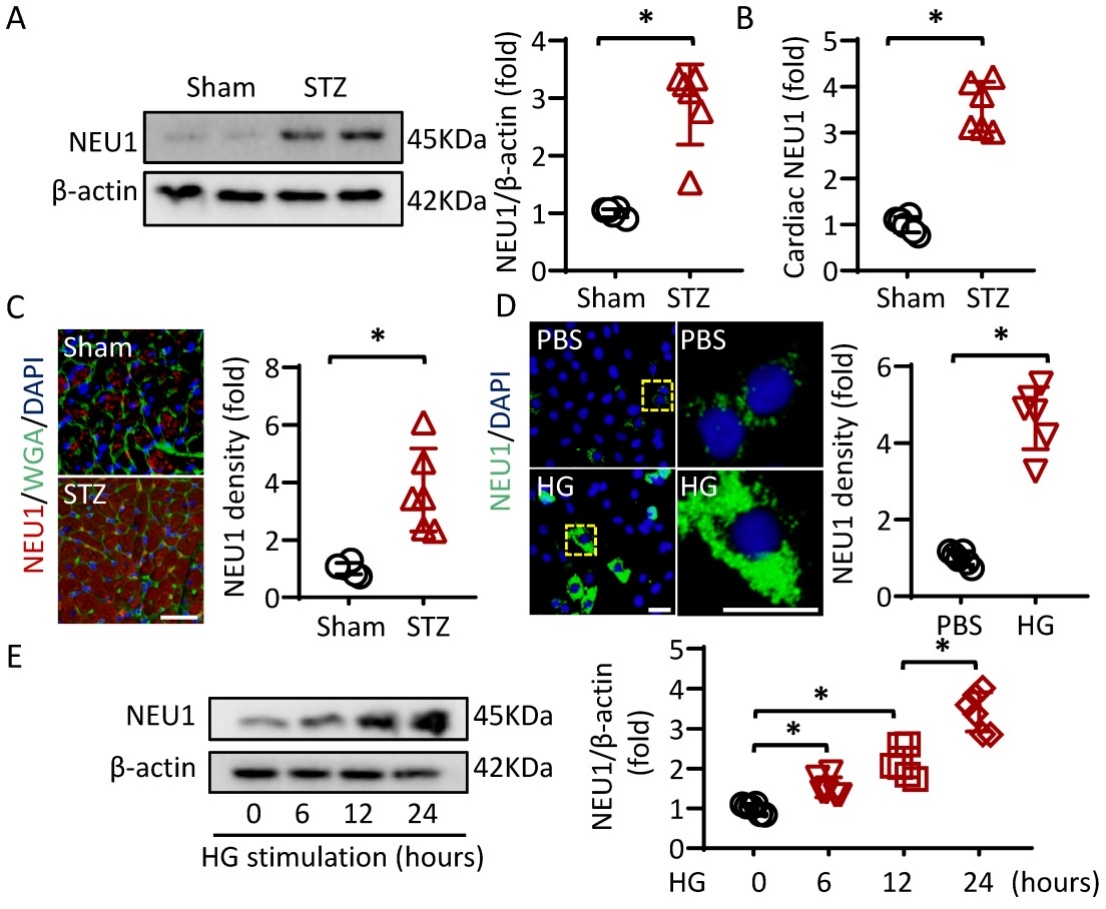
** NEU1 is elevated in STZ-induced diabetic mice heart and NRVMs incubated with high glucose.** A. Protein levels of NEU1 were determined by Western blot in heart samples from Sham and diabetic mice (n=6). B. Cardiac NEU1 expression detected by ELISA (n=6). C. Immunofluorescence with an anti-NEU1 antibody in slices from the indicated mice hearts (n=6). scale bar, 50μm. D. Immunofluorescence images of NEU1 in NRVMs with or without high glucose (n=6). scale bar, 50μm. E. Protein levels of NEU1 in NRVMs treated with high glucose for the indicated time (n=6). *P<0.05 vs. the matched group.

To investigate whether NEU1 is involved in the pathogenesis of diabetic cardiomyopathy, we first detected NEU1 expression levels by Western blot. The Results showed that NEU1 were significantly increased in the hearts of diabetic mice (Figure 1A). Furthermore, ELISA data confirmed that STZ treatment activated NEU1 expression in mice hearts (Figure 1B). Besides, the elevated NEU1 was mainly localized in cardiomyocytes based on co-localization with Wheat germ agglutinin (WGA) (Figure 1C). We further compared NEU1 expression in cardiomyocytes with or without high glucose. Consistently, the NEU1 protein expressions were markedly increased in the cardiomyocytes confirmed by immunofluorescence staining. Accordingly, time-dependent upregulation of NEU1 was observed in NRVMs treated with high glucose detected by Western Blot (Figure 1E).

### Diabetes-induced cardiac dysfunction is rescued by NEU1 inhibition

To investigate the role of NEU1 in STZ-induced diabetic cardiomyopathy, mice with AAV9 injection were exposed to STZ treatments. As shown in Figure S1A, AAV9-shNEU1#2 significantly inhibited NEU1 mRNA level in NRVMs comparing with others. The efficiency of AAV9-shNEU1 was presented in Figure S1B. Diabetic mice exhibited increased blood glucose levels (Figure S2A) with a decrease in body weight (Figure S2D). Diabetic mice also had increased glycosylated hemoglobin (HbA1c) levels with a concomitant decline in the pancreas insulin content (Figure S2B, S2C). While, knockdown NEU1 did not significantly alter the body weight/blood glucose level/pancreas insulin content neither in control nor in diabetic animals (Figure S2A-S2D).

**Figure 2:**
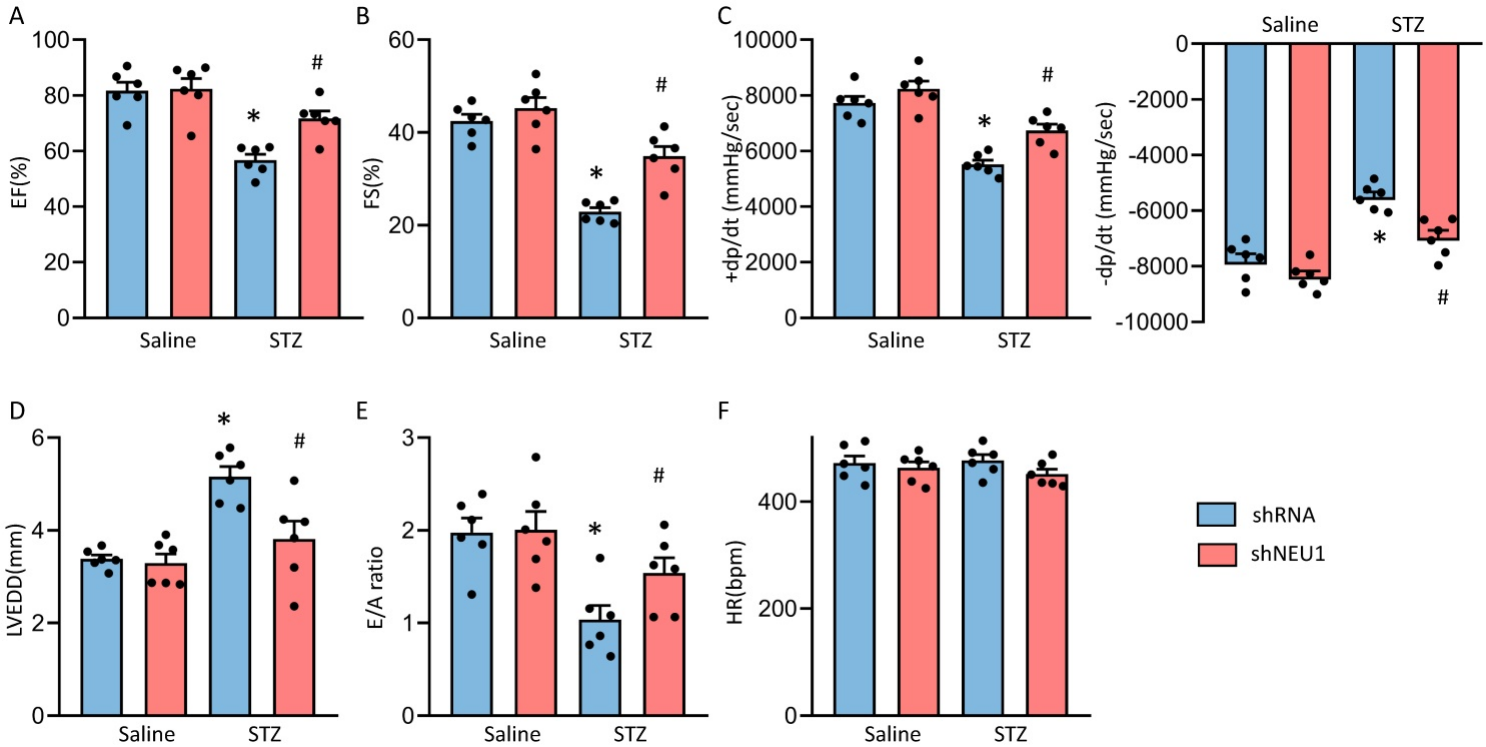
** NEU1 inhibition protected against diabetes-induced cardiac dysfunction in mice.** A-C. Cardiac function and hemodynamic parameters of mice (n=6). D. Left ventricle end-diastolic diameter (LVEDD) (n=6). E. E/A ratio (n=6). F. Heart rate (n=6). *P<0.05 vs. Saline+Vehicle group; ^#^*P*<0.05 vs. STZ+Vehicle group.

Next, we detected the alterations of cardiac function. Consistent with our previous research, mice developed deteriorated cardiac function, as assessed by increased left ventricular end diastolic diameter (LVEDD) and reduced fractional shortening (FS) and LV ejection fraction (LVEF)^13^. NEU1 knockdown rescued the deteriorated cardiac function, as reflected by LVEDD, LVEF and FS (Figure S2E and Figure 2A, B and D). NEU1 inhibition can improve cardiac systolic function (assessed by dP/dt max) and diastolic function (assessed by dP/dt min and E/A ratio) by pressure-volume analysis (Figure 2C and E). No significant difference in heart rate among these groups was observed (Figure 2F). However, AAV9-shNEU1 alone showed no beneficial effect on heart function under basal conditions (Figure 2). Generally, these data indicated that NEU1 inhibition could lead to alleviated cardiac dysfunction in STZ-induced mice.

### NEU1 inhibition alleviates cardiac fibrosis in STZ-induced diabetic mice

**Figure 3:**
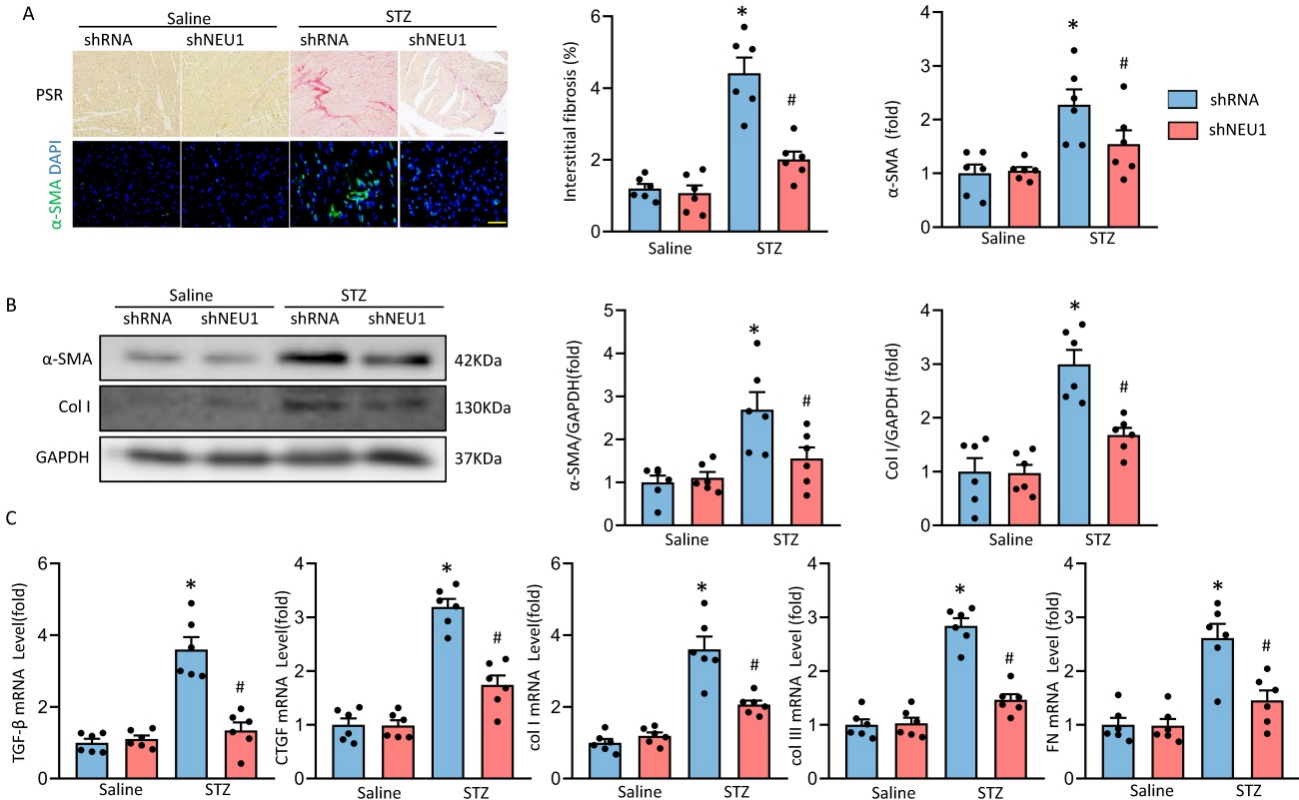
** NEU1 inhibition attenuates diabetes-associated myocardial fibrosis.** A. Representative images of the morphological analysis of cardiac fibrosis as reflected by PSR staining and immunofluorescence staining for a-SMA (bar=50 μm). Quantification of fibrotic areas (n=6). Quantitative results of a-SMA of immunofluorescence staining showing the activation of myofibroblasts (n=6). B. α-SMA and collagen I (col I) protein expression and quantitative data (n=6). C. The relative mRNA levels of TGF-β, connective tissue growth factor (CTGF), collagen I (col I), collagen III (col III), and fibronectin (FN) normalized to GAPDH in mice (n = 6). *P<0.05 vs. Saline+Vehicle group; ^#^*P*<0.05 vs. STZ+Vehicle group.

Next, we determined whether NEU1 inhibition could attenuate fibrosis in STZ-induced diabetic mice hearts. PSR-staining results showed that AAV9-shNEU1 mice presented with less collagen deposition than that in mice with AAV9-shRNA after STZ-induced, which was verified by the suppressed mRNA levels of fibrotic markers, such as connective tissue growth factor (*CTGF*), transforming growth factor-β (*TGF-β*), collagen I (*Col I*), collagen IIII (*Col III*) and fibronectin (*Fn*) (Figure 3A, 3C). Cardiac fibrosis was further verified by immunochemistry of α-smooth muscle actin (α-SMA) (Figure 3A). Also, NEU1 inhibition reduced the expression of α-SMA and *Col I* in STZ-induced diabetic mice (Figure 3B).

### Knockdown of NEU1 suppressed myocardial inflammation accumulation and apoptosis in diabetes heart

**Figure 4:**
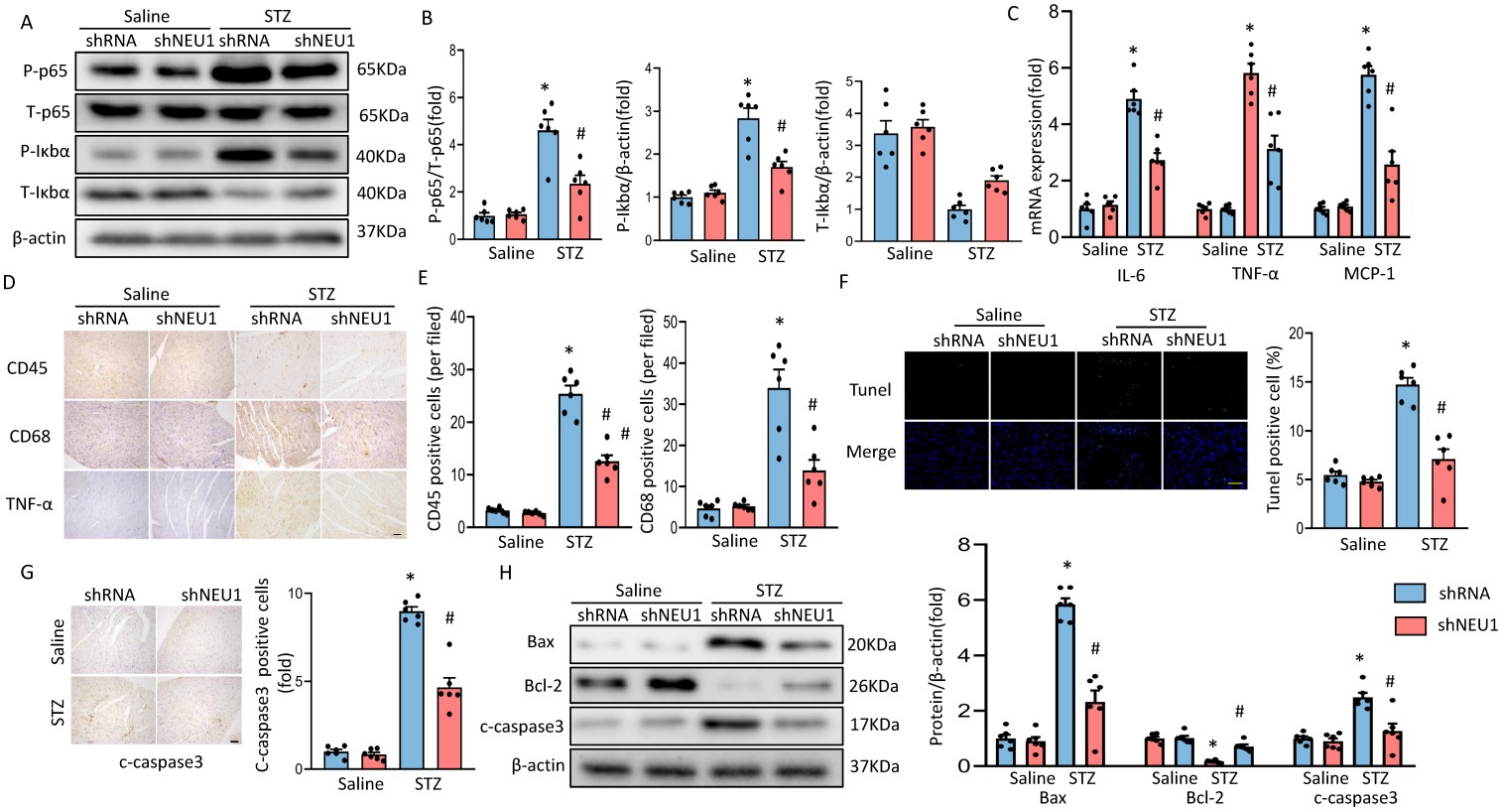
** NEU1 inhibition suppressed myocardial inflammation accumulation and apoptosis in diabetes heart.** A-B. Total and phosphorylated p65 and IκBα protein expression and quantitative data (n=6). C. The relative mRNA levels of IL-6, TNF-α, and MCP-1 normalized to GAPDH in mice (n=6). D-E. Representative images of immunohistochemistry analysis of CD45, CD68, and TNFα in diabetic hearts. Quantitative data (n=6). F. Myocardial apoptosis measured by TUNEL staining in heart sections (n=6, 10 fields per coverslip, bar=50 μm). G. Cleaved caspase-3 expression in the heart sections of the saline and diabetic mice determined by immunohistochemistry (n=6, 10 fields per coverslip, bar =50 μm). H. Western blot and quantitative analysis showing the protein levels of Bax, Bcl-2 and cleaved caspase-3 in vehicle and shNEU1 treated mice (n =6). *P<0.05 vs. Saline+Vehicle group; ^#^*P*<0.05 vs. STZ+Vehicle group.

As inflammation and apoptosis are the key features in the pathogenesis of diabetic cardiomyopathy. When upstream signal activates IκBα degradation, NF-κB p65 transfers from inactivated state and from cytoplasm to nucleus, binds to corresponding inflammation-related genes, initiates inflammatory cytokine transcription and induces inflammation 20. To determine the effect of shNEU1 on inflammation induced by STZ, we examined whether NEU1 inhibition affects inflammatory cell infiltration and NF-kB signaling in the heart. Western blot confirmed that the increase in the phosphorylation level of NF-κB family member p65 caused by STZ were inhibited by NEU1 inhibition (Figure 4A,4B). IκBα phosphorylation and IκBα degradation in vehicle-treated diabetic mice was significantly impaired in diabetic mice with NEU1 inhibition (Figure 4A,4B). Also, NEU1 knockdown can decreased the inflammatory response in STZ-induced diabetic mice hearts, as manifested by the decreased mRNA level of* IL-6*, *TNF-α* and *MCP-1* compared with the vehicle-treated diabetic group (Figure 4C). Furthermore, the infiltration of CD68-labeled macrophages and CD45-labeled leukocytes in diabetic mice increased significantly. In contrast, the migration and accumulation of inflammatory cells was alleviated by NEU1 inhibition (Figure 4D,4E). The results of TUNEL staining and immunohistochemical of cleaved caspase-3 revealed that NEU1 knockdown can inhibit apoptosis induced by STZ injection (Figure 4F, 4G). The protective effect on apoptosis was further confirmed by Western blot showing that NEU1 inhibition limited the alterations of the levels of BAX, C-caspase3 and BCL-2 level in diabetic hearts (Figure 4H).

### NEU1 inhibition attenuates diabetes-induced myocardial oxidative stress

**Figure 5:**
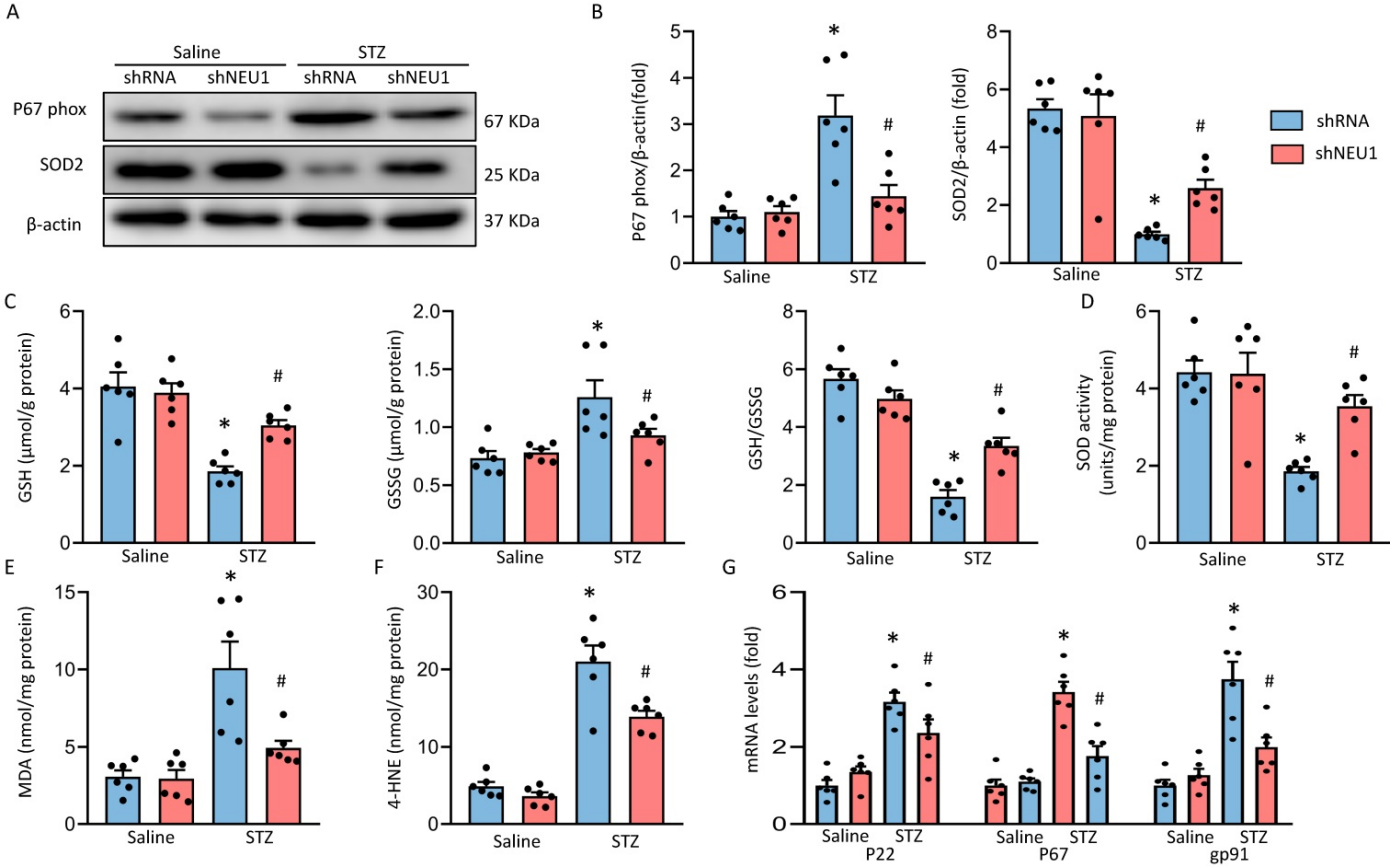
** shNEU1 attenuated diabetes-induced oxidative stress in the hearts.** A-B. Western blot and quantitative analysis showing the protein levels of p67 phox and SOD2 in vehicle and shNEU1 treated mice (n =6). C. Endogenous antioxidants (GSH) content (n=6). D. Total SOD activity in diabetic mice (n=6). E. Lipid peroxidation in diabetic hearts (n=6). F. 4-HNE (n=6). G. NADPH oxidase subunits mRNA expression by real time RT-PCR (n=6). *P<0.05 vs. Saline+Vehicle group; ^#^*P*<0.05 vs. STZ+Vehicle group.

As oxidative stress is the main feature of diabetic cardiomyopathy. Western blot results verified that NEU1 inhibition significantly decreased the P67phox and increased SOD2 expression in STZ-induced diabetic mice (Figure 5A, 5B). There was increased accumulation of GSSG with concordant decrease of GSH and GSH/GSSG ratio in heart of diabetic mice. While, these alternations were attenuated when mice treated with AAV9-shNEU1 in response to STZ injection (Figure 5C). Compared with mice in the control groups, total SOD activity was significantly reduced in diabetic mice and was increased by administration of AAV9-shNEU1 (Figure 5D). As shown in Figure 5E and 5F, NEU1 inhibition decreased lipid peroxidation in diabetic mice. Also, our results confirmed that the upregulation of P67*phox*, P22*phox* and gp91*phox* mRNA induced by STZ injection was reduced in diabetic mice with AAV9-shNEU1 administration (Figure 5G). Collectively, these data indicated that NEU1 inhibition exerted antioxidation effects in diabetic hearts.

### NEU1 promotes HG-induced cardiomyocyte injury by inhibiting SIRT3

**Figure 6:**
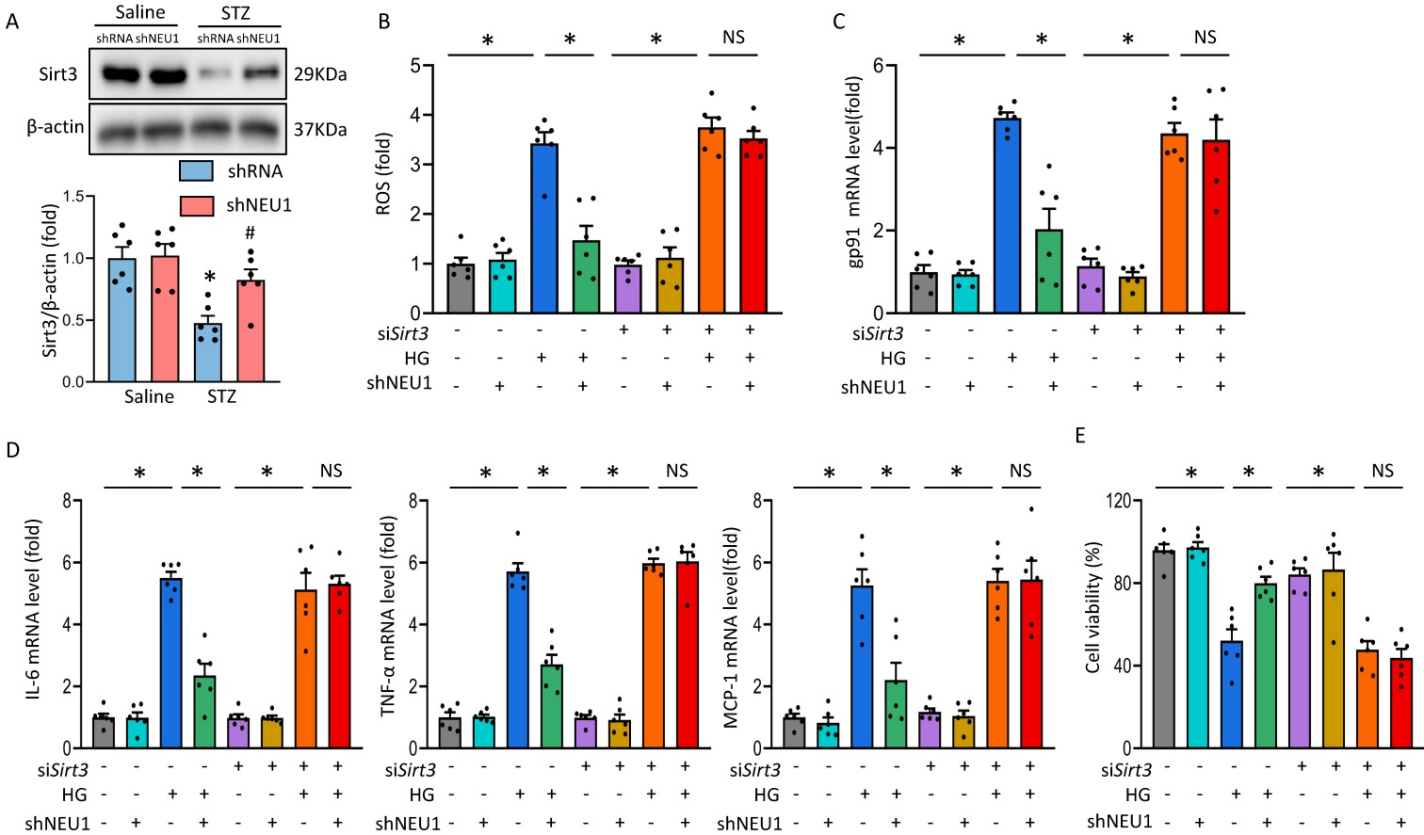
** NEU1 promotes HG-induced adverse changes via suppressing SIRT3 *in vitro.*** A. Western blot and quantitative analysis showing the protein levels of SIRT3 in vehicle and shNEU1 treated mice (n =6). *P<0.05 vs. Saline+Vehicle group; ^#^*P*<0.05 vs. STZ+Vehicle group. B. SIRT3 knockdown offset the protection afforded by NEU1 inhibition against ROS induced by HG (24h) (n=6). C. The relative mRNA levels of gp91 normalized to GAPDH under HG stimulation NRVMs (n = 6). D. The relative mRNA levels of IL-6, TNF-α, and MCP-1 normalized to GAPDH under HG stimulation in NRVMs (n= 6). E. Cell death was detected by an CCK8 assay under HG stimulation in NRVMs (n = 6). *P<0.05 vs. the matched group; NS indicates not significant.

It is reported that Sirtuin-3 (SIRT3) plays an important role in mitochondrial homeostasis and plays a protective role in the occurrence and development of DCM 21, 22. Consistent with previous studies^23^, we observed that STZ significantly inhibited the expression of SIRT3 at the level of mRNA and protein. Whereas, the inhibitory effect of STZ was counteracted by AAV9-shNEU1 administration (Figure 6A and Figure S3A.). Given the observation that NEU1 inhibition activated SIRT3 *in vivo*, we examined the role of SIRT3 in NEU1-mediated adverse effects *in vitro*. NRVMs were infected with siRNA knock down SIRT3 and the efficiency was confirmed by Western blot (Figure S3B). Accumulating evidences have shown that SIRT3 exerted cardiovascular protection roles by regulating oxidative stress 24. Intriguingly, administration of shNEU1 induced a significant reduction in ROS, a substantial decrease in mRNA of inflammatory mediators, and a significant increase in cell viability in NRVMs exposed to HG 24h. Consistent with the result *in vivo*, we found that the inhibitory effect of shNEU1 on ROS generation was blunted after the knock-down of SIRT3 (Figure 6B,6C and Figure S3C). Besides, NEU1 inhibition lost its effect on HG-induced inflammatory response in SIRT3-knockdown cells, as shown by the unaltered mRNA levels of IL-6, TNF-α and MCP-1 (Figure 6D). NRVMs exposed to HG for 72h had decreased cell viability, but the cell viability was increased when administration with shNEU1 (Figure 6E). HG also downregulated BCL-2 and upregulated BAX in NRVMs. However, these alternations are blocked by NEU1 inhibition (Figure 6E, Figure S3D). These effects of shNEU1 have been abolished by SIRT3 deficiency. Furthermore, H_2_O_2_ were used to induce myocyte apoptosis. SIRT3 knockdown completely abolished the protection by NEU1 inhibition against H_2_O_2_-induced apoptosis (Figure S3E). Consistent with the changes of molecules, we found that SIRT3 knock-down negated beneficial effects mediated by NEU1 inhibition on lipid peroxidation and oxidative damage in HG-treated NRVMs, as confirmed by the unaltered MDA, and SOD activity (Figure S3F). These results indicated that NEU1 promotes HG or STZ-induced oxidative stress, inflammatory response and cardiomyocytes apoptosis via downregulating the expression of SIRT3.

### AMPKα accounts for the key role of the SIRT3/SOD2 pathway *in vitro*

**Figure 7:**
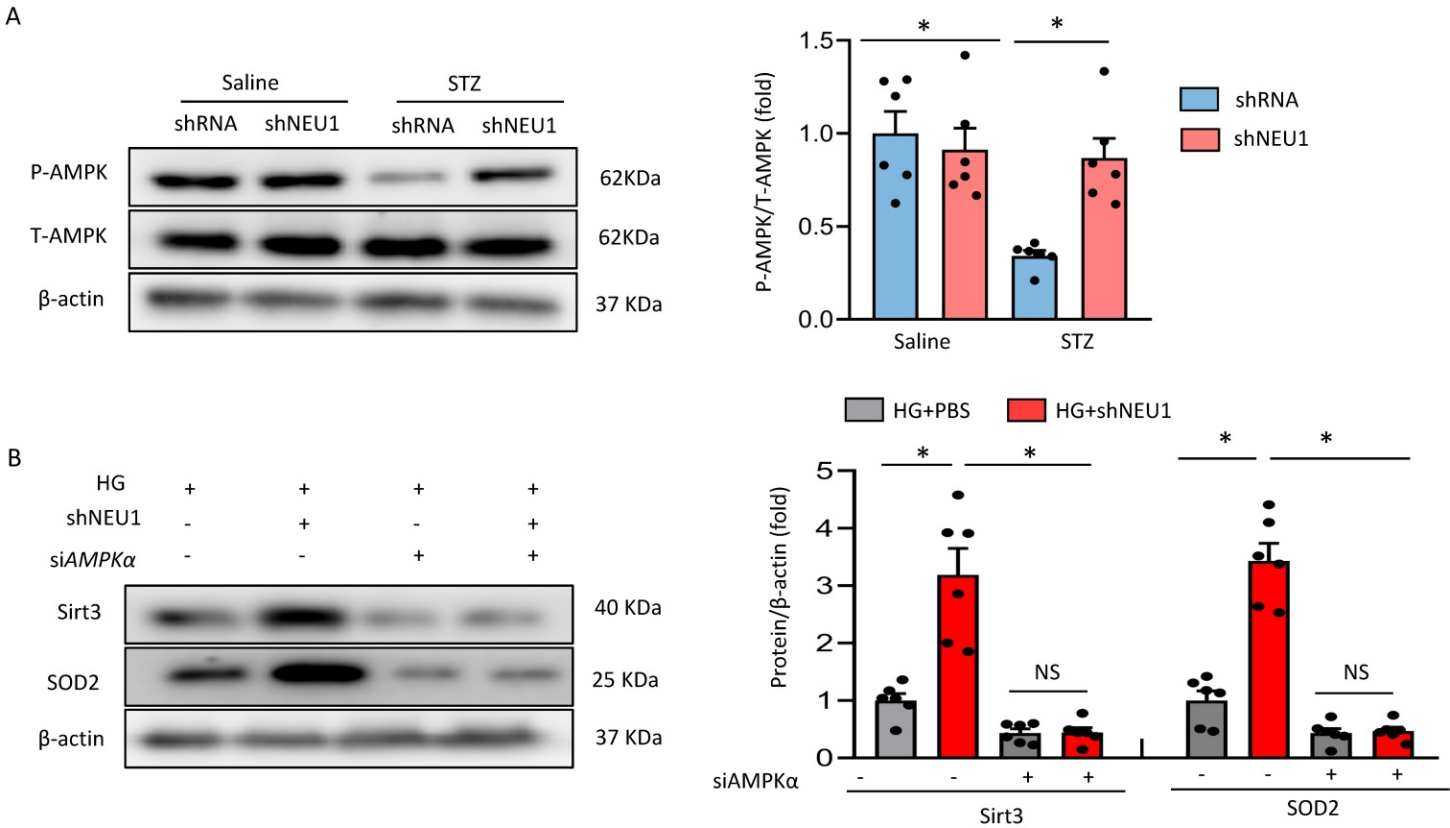
** AMPKα accounts for the key role of the SIRT3/SOD2 pathway.** A. Total and phosphorylated AMPKα protein expression and quantitative data (n=6). ^#^*P*<0.05 vs. STZ+Vehicle group. B. SIRT3 and SOD2 levels in NRVMs transfected with or without *si*AMPKα under HG stimulation (n=6). *P<0.05 vs. the matched group; NS indicates not significant.

Next, we discussed the possible mechanism of NEU1 promoting STZ-induced downregulation of SIRT3. Previous studied have found that the phosphorylation of AMPKα was decreased in diabetic cardiomyopathy. AMPK alleviates cardiac remodeling parameters and improves cardiac function through SIRT3/oxidative stress signal pathway 25. Hence, we supposed that NEU1 inhibition might prevent STZ-induced downregulation of SIRT3 via activating AMPKα. As expected, we found that shNEU1 treatment restored the reduction of AMPKα phosphorylation induced by HG in NRVMs (Figure 7A).To further prove the hypothesis that AMPKα was responsible for the upregulation of SIRT3, we knocked down AMPKα with siRNA, and the efficiency was evidenced by western blot (Figure S4A).Western blot results showed that AMPKα knockdown abrogated the restoration of SIRT3 and SOD2 level by NEU1 inhibition *in vitro* (Figure 7B).Consistent with the downregulation of SIRT3,we found that AMPKα knockdown significantly blocked the inhibitory effect of NEU1 inhibition on ROS accumulation and oxidative stress level (Figure S4B-S4C).In addition, the protective effect of NEU1 inhibition on inflammatory response and cell death was also blunted, as evaluated by the mRNA level of IL-6, TNF-α and cell viability (Figure S4D-S4F). Hence, we suggested that AMPKα accounts for the key role of SIRT3/SOD2 pathway.

### AMPKα deficiency offset the protective effects of shNEU1 *in vivo*

**Figure 8:**
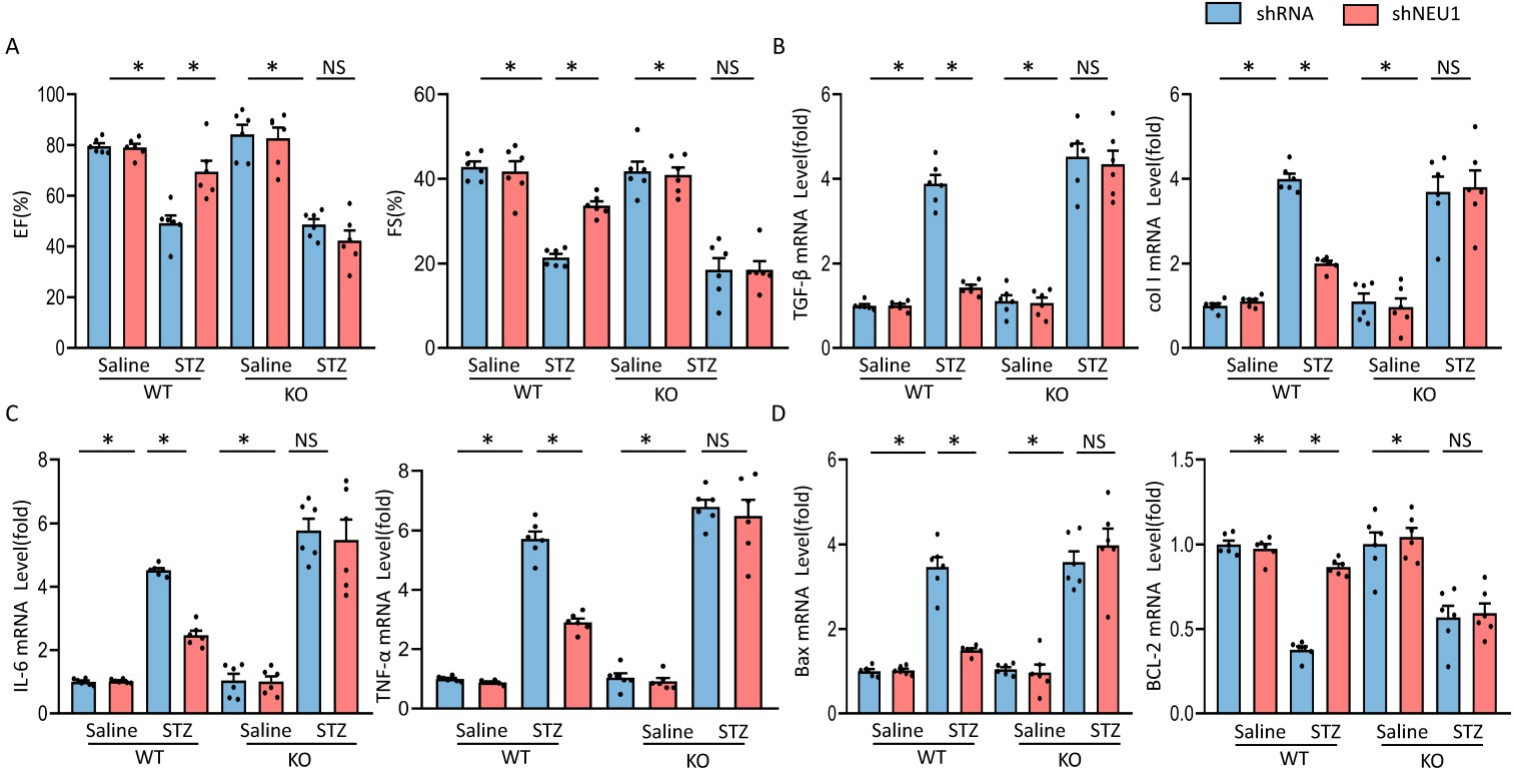
** AMPKα deficiency offset the protective effects of shNEU1 *in vivo.*** A. Heart function evaluated by echocardiography (n=6). B. mRNA levels of the fibrotic markers (n=6). C. mRNA levels of the inflammatory markers(n=6). D. mRNA levels of the apoptosis-related genes (n=6). *P<0.05 vs. the matched group; NS indicates not significant.

To further investigate whether the protective effects of NEU1 inhibition via AMPKα *in vivo*, AMPKα knockout (KO) mice were used. In line with the data *in vitro*, we observed that NEU1 inhibition lost the protective effect in AMPKα knockout mice, as evidenced by the indistinguishable LVEF and LVFS (Figure 8A). Besides, shNEU1-mediated downregulation of TGF-β and col I was all reversed by AMPKα deletion (Figure 8B). Consistent with the findings* in vitro*, shNEU1 lost its protective effects on inflammatory response in AMPKα-deficient mice, as reflected by the mRNA level of *TNF-α* and *IL-6* (Figure 8C). Furthermore, AMPKα KO abrogated the protective effects of NEU1 inhibition on cardiomyocytes apoptosis in response to STZ injection, as indicated by mRNA level of *BAX* and *BCL-2* (Figure 8D). Collectively, these data indicated that the protective of shNEU1 were dependent upon the activation of AMPKα.

### The precise mechanism of NEU1 regulating AMPKα

**Figure 9:**
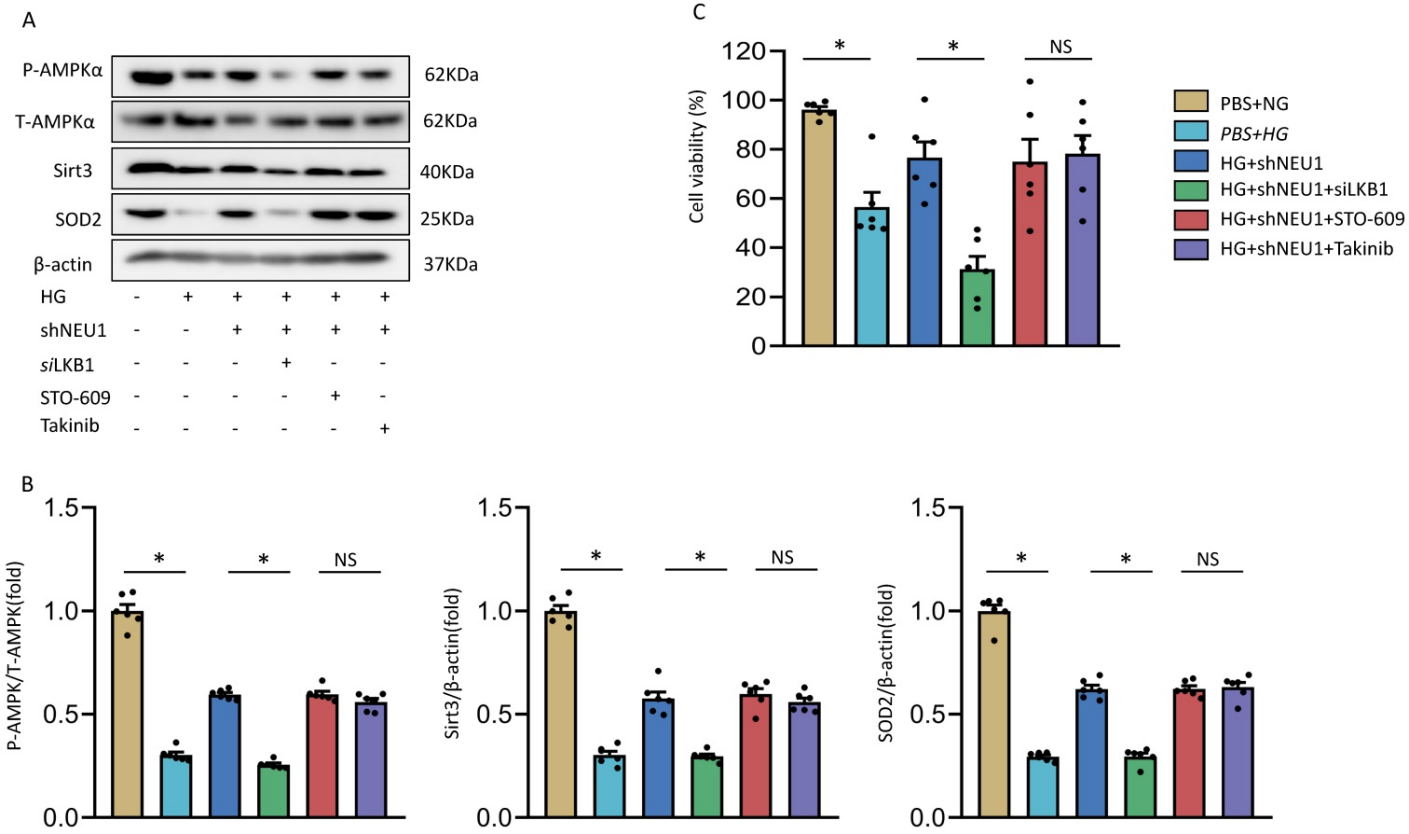
** The precise mechanism by which shNEU1 activates AMPKα.** A-B. p-AMPKα, SIRT3 and SOD2 levels in NRVMs treated with *si*LKB1, STO-609 (the CaMKK inhibitor, 800 nmol/l), Takinib (the selective TAK1 inhibitor, 20mmol/ml). C. Cell viability of NRVMs (n = 6). *P<0.05 vs. the matched group; NS indicates not significant.

At present, it has been found that there are three upstream kinases of AMPK, namely liver kinase B1(LKB1), transforming growth-factor-β-activated kinase-1(TAK1), Ca^2+^/calmodulin-dependent protein kinase kinase-β(CaMKKβ) respectively 26. We investigated the involvement of AMPK in the shNEU1-triggered activation of SIRT3 signaling. As shown in the previous experimental results, shNEU1 administration improved the phosphorylation level of the AMPKα and the expression level of SIRT3 and SOD2. Next, Takinib (a potent and selective TAK1 inhibitor), STO-609 (a selective CaMKKβ inhibitor) and small interfering RNA of LKB1(si*LKB1*) were used. As shown in Figure 9A and 9B, knockdown of LKB1 prevented shNEU1-triggered AMPKα activation. However, Takinib and STO-609 had no such effect on the activation of AMPKα (Figure 9A,9B). Corresponding to this change, after knocking down LKB1, the expression levels of SIRT3 and SOD2 did not increase after shNEU1 administration. Moreover, Takinb and STO-609 did not inhibit the expression of SIRT3 and SOD2 (Figure 9A ,9B). In addition, the protection effects of NEU1 inhibition against HG-induced cell loss was also interdicted by SiLKB1, but not Takinb and STO-609 (Figure 9C). Collectively, these results suggested that NEU1 might inhibits AMPKα via the suppression of LKB1.

## Discussion

**Figure 10:**
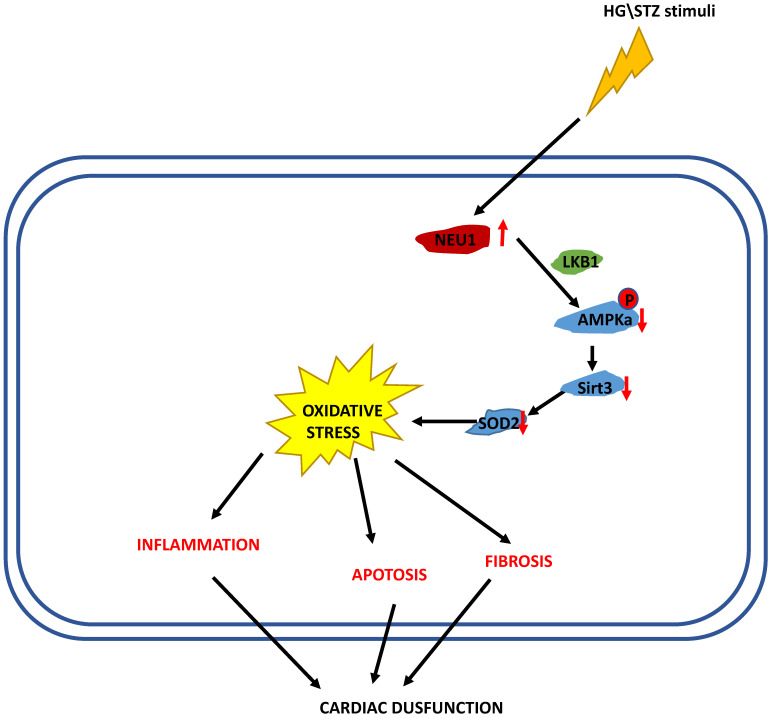
The proposed mechanism of NEU1 promotes diabetic cardiomyopathy

In the present study, we demonstrated that NEU1 inhibition alleviated deteriorated cardiac function in diabetic hearts, and protected against diabetes-related cardiac fibrosis, inflammation, oxidative damage and cell death *in vivo*. In this study, NEU1 inhibition also attenuated HG-induced production of ROS and inflammation, and reduced the cell loss induced by HG *in vitro*. Mechanistically, NEU1 inhibited AMPKα, and thus suppressed SIRT3 to promote subsequent oxidative damage, inflammatory response and cell lose in respond to the high glucose. We also found that NEU1 inhibition activated AMPKα via LKB1. Our data suggested that NEU1 inhibition exert its protective effect against diabetic cardiomyopathy via LKB1-AMPKα-SIRT3 pathway (Figure 10).

NEU1 is highly expressed in different types of immune cells, such as macrophages in atherosclerotic arteries, circulating monocytes and invading monocytes/macrophages in the heart 27, 28. It has been reported that NEU1 is involved in the development of inflammatory response and is involved in atherosclerosis and heart failure 29. In the mononuclear blood cells of patients with myocardial infarction, the expression of NEU1 increased compared with healthy controls. During carotid atherosclerosis, NEU1 is highly expressed in macrophages located in the intimal layer, calcified area, and outer membrane of the plaque 27. Isoproterenol can rapidly increase the sialidase activity of rat cardiomyocytes and cardiomyocyte-derived H9c2 cells 30. Recent studies have found that silencing or pharmacological inhibition of NEU1 prevented cardiac remodeling in response to pressure overload, inhibited cardiomyocytes hypertrophy and attenuated ischemia-related cardiac injury 10, 31. Despite their shared the cardioprotective effects of NEU1 deficiency in different cardiovascular diseases, our study contributes to supplement of the specific mechanism of NEU1 in the pathogenesis of diabetic cardiomyopathy. This work found that cardiomyocyte-localized NEU1 was increased significantly in STZ-induced diabetic mice heart and HG-stimulated cardiomyocytes. It is true that lowering blood glucose and increasing insulin sensitivity could improve the prognosis of diabetic cardiomyopathy. However, the aim of our experiment is mainly to explore the role of heart NEU1 in the process of diabetic cardiomyopathy. Therefore, we used myocardial injection of AAV9 to inhibit the expression of NEU1 in the myocardium. Moreover, AAV9-derived vectors displaying selective loss of liver tropism and demonstrating potential for cardiac and musculoskeletal gene transfer applications 32. Therefore, cardiac specific silencing NEU1 does not affect insulin sensitivity or hyperglycemia. This study combined with previous work suggests that NEU1 is a key driver of cardiovascular disease and is therefore a potential therapeutic target.

Studies have shown that the mitochondrial Sirtuin family is involved in insulin resistance in patients with diabetes 23, 33-35. SIRT3 can prevent or even reverse retina, bone and heart damage caused by diabetes. SIRT3-FOXO3A-Parkin signal pathway may play an important role in the occurrence and development of diabetic cardiomyopathy 36. Melatonin protects diabetic cardiomyopathy through MST1/SIRT3 signaling pathway 37, while garlic protects diabetic cardiomyopathy from oxidative stress by enhancing SIRT3 activity 38. In addition, APLN gene therapy can promote angiogenesis and improve the recovery of cardiac function in diabetic cardiomyopathy by up-regulating SIRT3 pathway 39. Obese mice fed with high-fat diet (HFD) had lower levels of SIRT3 in the heart, while mice with SIRT3 knockout had higher cardiac adipotoxicity 23, 33, 34. SIRT3 may regulate adipotoxicity by promoting lipid metabolism, reducing fatty acid accumulation and oxidative stress injury 40-42, and restoring cardiac remodeling function 33, 34. Based on the above research achievements, we detected SIRT3 expression in mice heart injected with STZ. As expected, the expression of SIRT3 decreased significantly *in vivo*. However, shNEU1 inhibited SIRT3 inactivation and significantly attenuated cardiac dysfunction and cardiac remodeling. In addition, knockdown of SIRT3 abrogated the beneficial effects *in vitro*. SIRT3 protects pancreatic cells from adipotoxicity by antagonizing cell damage induced by oxidative stress 43. With the increase of SIRT3 expression, SOD2 deacetylation decreases and SOD2 activity increases, this limits the accumulation of ROS 44. While it is true that SIRT3 depletion was linked to hyperacetylation of the key mitochondrial antioxidant, SOD2, leading to SOD2 inactivation and mitochondrial oxidative stress 45, 46. As prior study reported, SIRT3 directly deacetylates and activates mitochondrial isocitrate dehydrogenase 2 (Idh2), leading to increased NADPH levels and an increased ratio of reduced-to-oxidized glutathione in mitochondria 47. The elevated NADPH, in turn, is necessary for glutathione reductase, which converts GSSG into reduced glutathione GSH, the cofactor used by mitochondrial glutathione peroxidase (GPX) to detoxify ROS. Interestingly, another target of SIRT3, glutamate dehydrogenase (GDH). Moreover, mitochondrial ROS positively regulates NADPH oxidase subunits expression and activation under pathological conditions 48, 49. Therefore, SIRT3 regulate the expression of NAPDH oxidase via mitochondrial ROS.

Oxidative stress can induce abnormalities of calcium handling, which subsequently lead to diabetic cardiomyopathy and cardiac dysfunction 50. NEU1 plays an important role in cellular oxidative stress. Nrf2 activates the mechanical sensitivity of the endogenous antioxidant system is the key for ECs to adapt to oxidative stress in high shear stress areas. The down-regulation of endogenous sialidase NEU1 leads to imbalance of sialic acid and increased expression of Nrf2 target genes, directly involved in the regulation of Nrf2 signaling pathway by sialic acid through unidirectional shear stress. This leads to abnormal EC phenotypes and even atherosclerosis 51. Increases in ROS and inflammation promote interstitial collagen deposition, which is associated with interstitial fibrosis and impaired myocardial relaxation. Increasing the activity of *α-SMA*, *TGF-β* and functional decreased active matrix metalloproteinase-2 (*MMP-2*) play an important role in the development of cardiac fibrosis. Structural abnormalities associated with diabetic cardiomyopathy include cardiomyocyte necrosis, progressive abolition of muscular fibrils, collagen formation in connective tissue, and fibrosis 52. The data in our study suggested that NEU1 inhibition protected against diabetes-induced oxidative damage *in vivo* and *in vitro*.

In addition, we also found that high glucose-induced inflammation respond and apoptosis were prevented by shNEU1 administration, but promoted by SIRT3 deficiency *in vitro*. In addition to regulating oxidative stress, upregulation of NEU1 can promote the inflammatory invasion of monocytes/macrophages, enhance cardiomyocyte hypertrophy, weaken the function of gap junctions, and lead to heart failure. Increased NEU1 expression in cells can promote the expression of inflammatory factors (TNF-α, IL-1, IL-6, etc.). NEU1 has been reported to be involved in the expression of TLRs and the regulation of downstream signaling pathways 53, 54. In human T cells, both NEU1 and NEU3 mRNA can be significantly induced by T cell receptor stimulation, and some cytokines, including interleukin (IL)-2 and IL-13, can be induced by the up-regulation of these salivary enzymes 55. These anti-inflammatory and anti-oxidative actions might explain the cardioprotection of shNEU1 on diabetic mice. Cardiac cell loss is one of the main factors that resulted in the impairment of cardiac function. Here, we also found that cells apoptosis in diabetic hearts was attenuated by NEU1 inhibition but aggravated by SIRT3 deficiency. Here we found that shNEU1 increased SIRT3 expression *in vivo* and *in vitro*. Our studies suggest that the elevated of SIRT3 following NEU1 inhibition is the primary mechanism leading to limited pathophysiological changes result in oxidative stress, immune responses, fibrosis, apoptosis and cardiac dysfunction.

Previous studies have found that AMPK plays a key role in intracellular metabolism and is an attractive therapeutic target, and that switching on/off of AMPK will leads to changes in the expression of SIRT3^56^. There is currently no research on the link between NEU1 and AMPK. In agreement with this finding, we found that AMPKα deficiency abolishes the protection provided by shNEU1 against fibrosis, inflammation, apoptosis and oxidative stress. Our study further suggested that the inhibitory effects of shNEU1 against diabetes-related cardiac dysfunction were offset by AMPKα deficiency *in vivo*. Importantly, shNEU1 elevated SIRT3 expression was inhibited by AMPKα deficiency, declaring that AMPKα activation was responsible for shNEU1-mediated upregulation of SIRT3.

A critical issue raised by our data in this study relates to the activation of the AMPKα pathway by NEU1 inhibition. AMPK activation is mediated by upstream kinases (such as LKB1, CaMMKβ, and TAK1)^26^. In this study, we found that si*LKB1* blocked shNEU1-induced AMPKα activation. However, the observation that CaMMKβ inhibitor and TAK1 inhibitor did not affect AMPKα activation caused by shNEU1 suggested that CaMMKβ and TAK1 were not involved in the shNEU1-mediated AMPKα activation. These results further indicate that NEU1 inhibition activates AMPKα via LKB1 on diabetic cardiomyopathy. It has been reported that AMPK is activated through phosphorylation via LKB1 at the lysosome in an AMP-independent manner. LKB1 which was required for canonical energy sensing by AMPK 57. We do not yet know the mechanisms of NEU1 on LKB1 activity; however, this determination will require extensive work that is beyond the scope of our study. The cellular targeting of LKB1 in the NRVM infected with shNEU1 is adequate for the points made in this manuscript: that the cell viability after high glucose is rescued, at least in part, by NEU1 inhibition, and that NRVM with LKB1 silence but not CaMMKβ inhibitor and TAK1 inhibiter, blocked the NEU1 inhibition effects in cell viability, as well as the expression of downstream signaling.

This study has some limitations. First, NEU1 was elevated in STZ-induced diabetic mice heart and in HG-stimulated cardiomyocytes. However, we did not explore why and how NEU1 increased, nor did we explore whether its expression in other tissues or cells changed, such as pancreatic islets. Second, we found that silencing NEU1 can activate AMPK in a high glucose environment through LKB1, but we have not further studied the specific mechanism by which NEU1 directly affects LKB1. Third, in this experiment, we mainly used AAV9 to verify its effect, and did not use genetically engineered mice related to NEU1. Fourth, in this study, only the effects of NEU1 were tested, and did not verify the expression and role of other NEUs (eg. NEU3). Fifth, this research did the exploration of NEU1 inhibition, but did not conduct the research of overexpression of NEU1.

In summary, the present study identified NEU1 deficiency attenuated STZ-induced diabetic cardiomyopathy. NEU1 inhibition activates AMPKα via LKB1, and subsequently activates sirt3, thereby regulating fibrosis, inflammation, apoptosis and oxidative stress in diabetic myocardial tissue.

## Supplementary Material

Supplementary figures and tables.
