# Supplementary material for: Neuraminidase 1 deficiency attenuates cardiac dysfunction, oxidative stress, fibrosis, inflammatory via AMPK-SIRT3 pathway in diabetic cardiomyopathy mice
Source: Int J Biol Sci. 2022 Jan 1;18(2):826–40. doi: 10.7150/ijbs.65938 (PMC8741837; doi:10.7150/ijbs.65938)

**Data supplement**

**Neuraminidase 1 deficiency attenuates cardiac dysfunction, oxidative stress, fibrosis, inflammatory via AMPK-Sirt3 pathway in diabetic cardiomyopathy mice**

Zhen Guo[1,2]*, Hu Tuo[1,2]*, Nan Tang[3,4]*, Fang-Yuan Liu[1,2]*, Shu-Qing Ma[1,2], Peng An[1,2], Dan Yang[1,2], Min-Yu Wang[1,2], Di Fan[1,2#], Zheng Yang[1,2#], Qi-Zhu Tang[1,2#]

**Supplementary Table 1: The information of the primary antibodies used in western blot**

| Antibody description | Company | No. and dilution |
|---|---|---|
| NEU1 | Abcam | #ab233119; 1:1000 |
| αSMA | Abcam | #ab5694; 1:1000 |
| Collagen I | Abcam | #ab34710; 1:1000 |
| β-actin | Abcam | #ab8227, 1:1000 |
| P-P65 | Bio-world | #BS4135, 1:1000 |
| T-P65 | Cell Signaling Technology | #8242, 1:1000 |
| P-IκBα | Abcam | #ab133462, 1:1000 |
| T-IκBα | Abcam | #ab7217, 1:1000 |
| Bax | Cell Signaling Technology | #2722, 1:1000 |
| Bcl-2 | Cell Signaling Technology | # 2870, 1:1000 |
| c-caspase3 | Cell Signaling Technology | #9661, 1:1000 |
| P67 phox | Abcam | #ab109366, 1:1000 |
| SOD2 | Abcam | #ab13533, 1:1000 |
| SIRT3 | Abcam | #ab189860, 1:1000 |
| P-AMPKα | Cell Signaling Technology | #2535, 1:1000 |
| T-AMPKα | Cell Signaling Technology | # 2603P, 1:1000 |

**Supplementary Table 2: The information of the primers used in RT-PCR.**

| Target | Forward Sequences (5'→3') | Reverse Sequences (5'→3') |
|---|---|---|
| Mice-GAPDH | TCATCAACGGGAAGCCCATC | CTCGTGGTTCACACCCATCA |
| Mice-TGFβ1 | ATCCTGTCCAAACTAAGGCTCG | ACCTCTTTAGCATAGTAGTCCGC |
| Mice-CTGF | CTCGTGGTTCACACCCATCA | AGTTGGCTCGCATCATAGTTG |
| Mice-Col1 | CCCAACCCAGAGATCCCATT | GAAGCACAGGAGCAGGTGTAGA |
| Mice-Col3 | GATCAGGCCAGTGGAAATGT | GTGTGTTTCGTGCAACCATC |
| Mice-fibronectin | CCGGTGGCTGTCAGTCAGA | CCGTTCCCACTGCTGATTTATC |
| Mice-IL6 | GTCAGCTGGATAGCGACA | GAAGCACAGGAGCAGGT GTAGA |
| Mice-TNFα | GACATGCCGCCTGGAGAAAC | AGCCCAGGATGCCCTTTAGT |
| Mice-MCP1 | TGGCTCAGCCAGATGCAGT | CCAGCCTACTCATTGGGATCA |
| Mice-p67phox | GCCGGAGACGCCAGAAGAGCTA | GGGGCTGCGACTGAGGGTGAA |
| Mice-gp91phox | TTCCAGTGCGTGTTGCTCGACA | TGGCGGTGTGCAGTGCTATCAT |
| Mice-SIRT3 | GGTCCGGGAGTGTTACAGG | CACCATGACCACCACCCTAC |
| Mice-Bax | CTGGATCCAAGACCAGGGTG | CCTTTCCCCTTCCCCCATTC |
| Mice-BCL2 | CTGAGTACCTGAACCGGCAT | GGTATGCACCCAGAGTGATG |
| Rat-GAPDH | GACATGCCGCCTGGAGAAAC | AGCCCAGGATGCCCITTAGT |
| Rat-p67phox | TTCCATCCCCAAATGCAAAG | TCAGATGCCCTAAAACCGGAG |
| Rat-gp91phox | GACCATTGCAAGTGAACACCC | AAATGAAGTGGACTCCACGCG |
| Rat-IL6 | GTTGCCTTCTTGGGACTGATG | ATACTGGTCTGTTGTGGGTGGT |
| Rat-TNFα | AGCATGATCCGAGATGTGGAA | TAGACAGAAGAGCGTGGTGGC |
| Rat-MCP1 | AGTCGGCTGGAGAACTACAAGA | CTGAAGTCCTTAGGGTTGATGC |

**Figure S1. NEU1 expression after administrated with AAV9-shRNA or AAV9-shNEU1**

A. mRNA levels of NEU1 after administrated with AAV9-shRNA or AAV9-shNEU1 in NRVMs (n=6). B. NEU1 protein expression and quantitative data (n=6).

**Figure S2. NEU1 inhibition did not affect blood glucose level and body weight in diabetic mice**

A. Blood glouse of mice at duration of diabetes (n=6). B. Pancreas insulin of mice at duration of diabetes (n=6). C. Glycosylated hemoglobin of mice at duration of diabetes (n=6). D. Body weight at the initial and end of study (n=6). E. Representative image of echocardiography. NS indicates not significant.

**Figure S3. ShNEU1 exerted its cardiac protection via activating Sirt3 in vitro**

A. mRNA levels of SIRT3 in vehicle and shNEU1 administered mice (n =6). *$P$<0.05 vs. saline+vehicle group; $^{\#}P$<0.05 vs. stz+vehicle group. B. Western blot for sirt3 and the quantitative data for NRVMs (n= 6). C. The relative mRNA levels of p67*phox* and p22*phox* normalized to GAPDH under HG stimulation in NRVMs (n = 6). D. The relative mRNA levels of BAX and BCL-2 normalized to GAPDH under HG stimulation in NRVMs (n = 6). E. Cell death was detected by an CCK8 assay under $H_2O_2$ stimulation in NRVMs (n = 6). F. The content of MDA and SOD activity under HG stimulation in NRVMs (n=6). *$P$<0.05 vs. the matched group; NS indicates not significant.

**Figure S4. Knockdown of AMPKα blunted the protective effects of shNEU1 in vitro.**

A. Western blot for AMPKα and the quantitative data in NRVMs (n= 6). B-C. The ROS level and SOD activity under HG stimulation in NRVMs (n=6). D-E. The relative mRNA levels of IL-6 and TNF-α normalized to GAPDH under HG stimulation in NRVMs (n = 6). F. Cell death was detected by an CCK8 assay under HG stimulation in NRVMs (n = 6). *$P$<0.05 vs. the matched group; NS indicates not significant.

**Figure S1.**

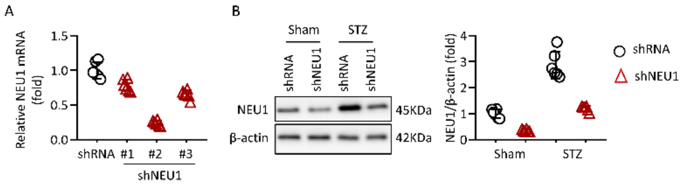

A
B
Relative NEU1 mRNA (fold)
1.5
1.0
0.5
0.0
shRNA
#1
#2
#3
shNEU1
Sham
STZ
shRNA
shNEU1
shRNA
shNEU1
NEU1
β-actin
45KDa
42KDa
NEU1/β-actin (fold)
4
3
2
1
0
Sham
STZ
shRNA
shNEU1

**Figure S2.**

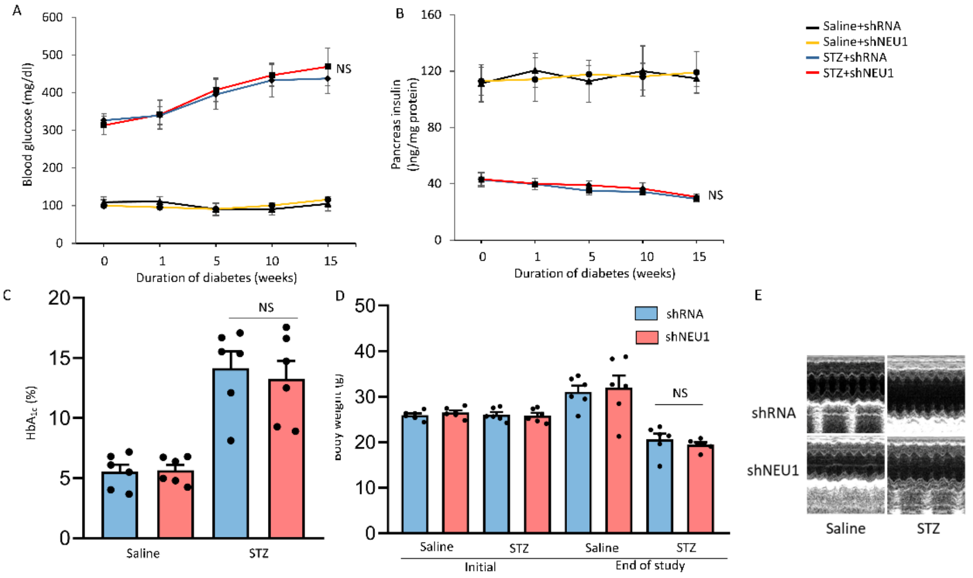

A
Blood glucose (mg/dl)
Duration of diabetes (weeks)
NS
B
Pancreas insulin
()ng/mg protein)
Duration of diabetes (weeks)
NS
Saline+shRNA
Saline+shNEU1
STZ+shRNA
STZ+shNEU1
C
HbA1c (%)
NS
Saline
STZ
D
shRNA
shNEU1
NS
Saline
STZ
Saline
STZ
Initial
End of study
E
shRNA
shNEU1
Saline
STZ

**Figure S3.**

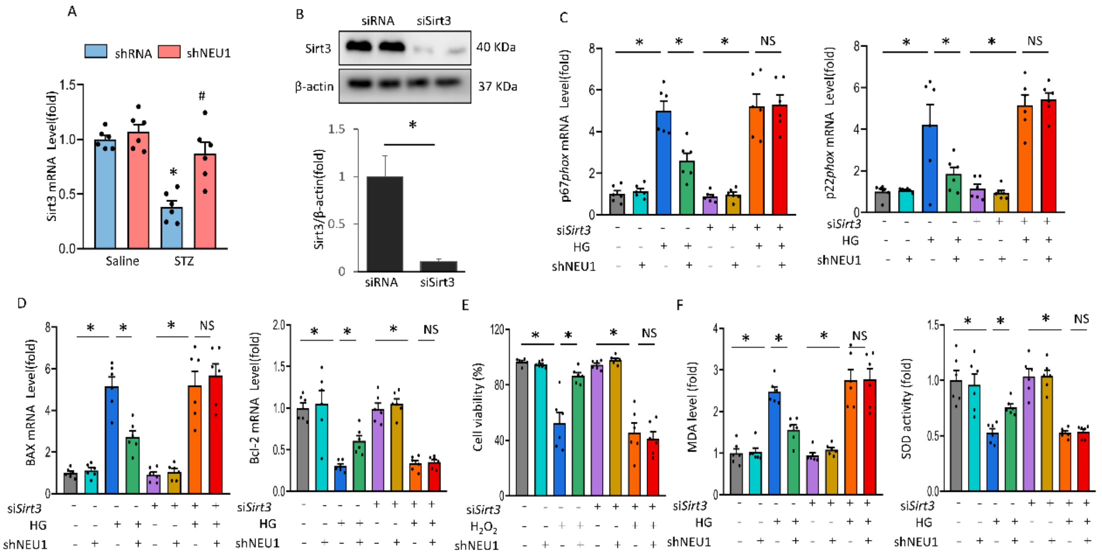


**Figure S4.**

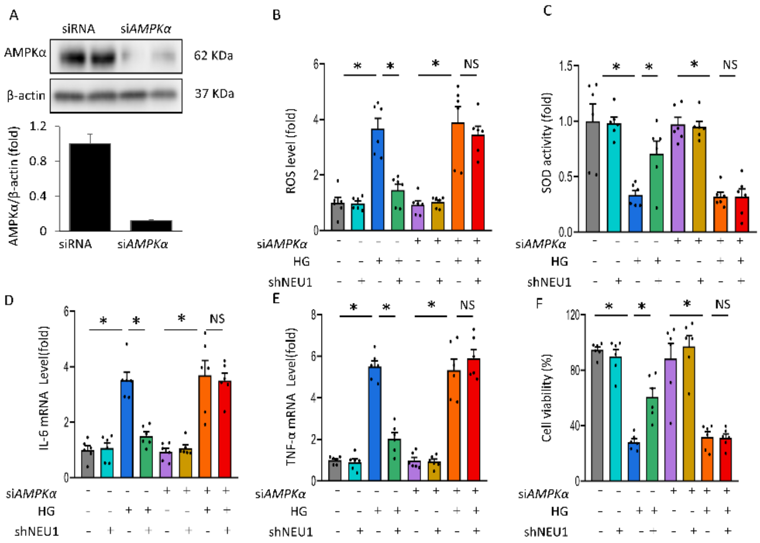

Supplement: Supplementary file 1 — Supplementary figures and tables. [file ijbsv18p0826s1.pdf]
